# The Physicochemical, Sensory, and Functional Properties of Yogurt Containing Millet and Milk

**DOI:** 10.3390/foods14203491

**Published:** 2025-10-14

**Authors:** Hui Wang, Yingyu Zhang, Yuxuan Han, Jiaxin Hou, Yingjun Zuo, Yan Li, Hua Wu

**Affiliations:** 1 Beijing Engineering and Technology Research Centre of Food Additives, School of Food and Health, Beijing Technology and Business University, Beijing 100048, China; 15531725502@163.com (H.W.); 13146870257@163.com (J.H.); wxsxhzhn@163.com (Y.Z.); 2 School of Light Industry Science and Engineering, Beijing Technology and Business University, Beijing 100048, China; z2330402143@163.com (Y.Z.); 13021115218@163.com (Y.H.)

**Keywords:** yogurt, millet, co-fermentation, antioxidant activity, anti-inflammatory activity

## Abstract

With growing consumer demand for functional dairy products, developing yogurts enriched with natural bioactive ingredients has become a research focus. Millet, a traditional cereal rich in polyphenols and dietary fiber, remains understudied in fermented dairy applications. This study evaluated the physicochemical properties, sensory quality, and functional activities of yogurt co-fermented with millet. Millet liquid, pre-treated through gelatinization and α-amylase liquefaction, was co-fermented with milk at addition ratios of 40% and 60% (*w*/*w*). The results indicated that millet liquid increased *Lactobacillus delbrueckii* subsp. *Bulgaricus* viability (8.55–8.58 log CFU/g vs. 8.26 log CFU/g in the control), improved viscosity (up to 1.0–1.6-fold higher than the control), enhanced texture properties (51–65-fold increase in springiness, 4.3–4.6-fold higher chewiness), and reduced syneresis (18.6–49.2% lower than the control). Sensory evaluation revealed superior flavor and sweetness in millet-enriched yogurt, achieving significantly higher scores than plain yogurt (*p* < 0.05). Functionally, the 60% millet yogurt showed 77.8% and 84.3% higher DPPH and ABTS radical scavenging capacities, respectively. Additionally, it suppressed DSS-induced inflammatory cytokine secretion in Caco-2 cells (27.2–69.7% inhibition of TNF-α, IL-6, and IL-1β). The improved antioxidant and anti-inflammatory activities may be attributed to polyphenol release from millet. This work highlights the potential of millet–milk co-fermentation for developing yogurts with enhanced texture, sensory appeal, and bioactive properties.

## 1. Introduction

The growing consumer demand for functional dairy products has resulted in the development of various innovative yogurt products with specific functionalities. These include the incorporation of natural functional ingredients rich in phenolic compounds, flavonoids, and anthocyanins to enhance the antioxidant, antidiabetic, anti-obesity, anti-inflammatory, antibacterial, and anticancer properties of yogurt [1,2,3,4,5].

Cereals are rich in dietary fiber and are essential in daily nutritional regimes. In recent years, an increasing number of cereal products rich in phenolic compounds and dietary fiber have been added to dairy products to improve their functionality. Several studies have found that adding wheat and rice bran to yogurt increased the probiotic count, likely attributable to the starchy, nitrogenous properties of these brans, as well as the presence of structural polysaccharides, such as β-glucan, with prebiotic characteristics [6,7,8]. Studies showed that supplementing yogurt with dietary fiber extracted from cereals, such as β-glucan, improved its texture and sensory qualities. This was attributed to the interaction between β-glucan and casein, which enhanced the protein network structure [9,10]. In addition, phenolic-rich cereals, such as rice and Tartary buckwheat, enhanced the antioxidant activity of yogurt and attenuated H_2_O_2_-induced cytotoxicity [11,12].

However, the potential of millet, a traditional cereal in many countries, such as China, India, and Afghanistan, for use in fermented dairy products remains underdeveloped [13]. Millet offers an abundance of nutritional components, including protein, starch, fats, vitamins, minerals, and a high crude fiber content [14], as well as phytochemicals, such as phenolics and carotenoids. Millet contains several crucial phenolic compounds, including a variety of phenolic acids and flavonoids with notable antioxidant capacity [15]. Millet consumption offers numerous health benefits, including a reduced risk of multiple chronic diseases, such as abnormal cholesterol metabolism, hypertension, and type 2 diabetes [16,17]. Many studies have shown that millet flour can be used as an ingredient in baked products, such as cakes and cookies, as well as a binding agent in meat products, including sausages, meatballs, and chicken patties [18,19,20,21,22].

Existing studies on grain-fortified yogurt have focused on texture improvement and probiotic activity enhancement. Based on this, this study innovatively introduced α-amylase and glucoamylase into the millet-supplemented yogurt fermentation system to optimize the glucose supply by degrading starch to promote LAB metabolism. This strategy naturally enhances the sweetness of the product

In this study, yogurt was prepared by co-fermentation of α-amylase-liquefied millet liquid (40% and 60% *w*/*w*) with milk. Furthermore, the physicochemical properties, lactic acid bacterial counts, sensory characteristics, and in vitro antioxidant activity of the millet yogurt were investigated. Cell-based in vitro models were employed to assess the effect of millet yogurt on DSS-induced inflammatory factor expression. The results of this study provide a theoretical basis and technical reference for the development of new yogurt with both nutritional and functional properties.

## 2. Materials and Methods

### 2.1. Materials

Whole milk powder was purchased from Fonterra Commercial Trading Co., Ltd. (Shanghai, China), which was made from the milk of Holstein cows. Jingu 21 millet was obtained from Jinzhong Fengyuan Planting Professional Cooperative (Jinzhong, Shanxi, China). Jingu 21 is a high-yielding foxtail millet cultivar developed by the Shanxi Academy of Agricultural Sciences, China. In its name, “Jin” refers to Shanxi Province (abbreviated as “Jin” in Chinese) and “No. 21” denotes the breeding sequence of the variety. Food-grade α-amylase and glucoamylase were supplied by XiaSheng Biotechnology Development Co., Ltd. (Beijing, China). Yogurt starter culture YC-381, containing *Streptococcus thermophilus* and *Lactobacillus delbrueckii* subsp. *bulgaricus*, was obtained from Chr. Hansen (Hoersholm, Denmark). Maltodextrin (food grade) was purchased from Samyang Genex (Gwangju, Republic of Korea).

### 2.2. Preparation of the Millet Liquid

The millet was crushed using a multi-functional pulverizer (FT-3200B, Fangtai Electric Co., Dongguan, China) and sieved through an 80-mesh stainless steel sieve (pore size: 0.177 mm, Zhentai Machinery, Xinxiang, China) to obtain millet flour. Subsequently, based on pre-optimization experiments, millet flour was mixed with distilled water at a ratio of 1:3 and soaked at 50 °C for 30 min. After gelatinization at 90 °C for 60 min, α-amylase (0.3 g/kg of millet flour) was added, and the mixture was treated at 80 °C for 90 min according to the amylase instruction manual (liquefaction stage), after which it was filtered through a 200-mesh sieve to obtain the millet flour liquid (MFL).

The millet was mixed with distilled water at a ratio of 1:3, pulped using a blender (L18-Y928S, Joyoung Bean Products Co., Ltd., Hangzhou, China), and soaked for 30 min at 50 °C. After gelatinization at 90 °C for 60 min, α-amylase (0.3 g/kg of millet) was added, and the mixture was treated at 80 °C for 90 min, filtered through an 80-mesh sieve, and then passed through a 200-mesh sieve to obtain the millet pulp liquid (MPL).

### 2.3. Yogurt Production

Table 1 shows the individual yogurt sample formulations. The whole milk powder was compounded in distilled water with the MFL, the MPL (40% and 60%), and maltodextrin, after which each sample was preheated to 60 °C and homogenized at 20 MPa. The mixtures were heated at 95 °C for 5 min, cooled to 40–42 °C, and inoculated with glucoamylase and a starter culture. The inoculated milk was fermented in a thermostatic incubator (LRH-250F, Shanghai Yiheng Scientific Instrument Co., Shanghai, China) at 42 °C ± 0.5 °C until reaching a pH value of 4.6. The yogurt was rapidly cooled in an ice-water bath, stirred, and cooled at 4 °C for 12 h. The yogurt samples were stored at 4 ± 1 °C for further analysis.

### 2.4. Physicochemical Determinations

#### 2.4.1. Basic Composition

The total solid content of millet liquid was determined according to the AOAC method. Samples were placed in a forced-air drying oven (Model: DHG-9070A, Shanghai Yiheng Scientific Instrument Co., Ltd., Shanghai, China) and dried at 105 °C ± 1 °C for 24 ± 2 h until reaching constant weight [23]. The protein level was measured using the Kjeldahl method with a conversion factor of 5.83 for millet [24]. The soluble solids in the millet liquid were determined using a refractometer (MASTER-M, Atago, Tokyo, Japan).

#### 2.4.2. pH and Titratable Acidity (TA)

The pH values of the yogurt samples were measured using a glass electrode pH meter (FE20, Mettler Toledo, Zurich, Switzerland), while the TA (%) was determined according to the GB 5009.239-2016 Chinese national standard [25].

#### 2.4.3. Determination of Syneresis

Syneresis was assessed using a previously described method [26]. Here, 25 g of yogurt was centrifuged at 2900× *g* and 4 °C for 15 min, after which the supernatants (whey) were weighed. Syneresis was calculated using the following equation:

(1) $$\mathrm{Syneresis}\;\left(\%\right)=\frac{\mathrm{Weight}\;\mathrm{of}\;\mathrm{whey}\;(g)}{\mathrm{Weight}\;\mathrm{of}\;\mathrm{yogurt}\;(g)}\times 100$$

#### 2.4.4. Textural Analysis

The texture of the yogurt was assessed using a previously described method with some modifications [27]. A 60 mL yogurt sample was placed in a 100 mL plastic container and used to assess the yogurt texture via a texture analyzer (TMS-Pro, Food Technology Corporation, Rockland, MA, USA) equipped with a 38.1 mm diameter cylindrical probe. The testing parameters included a contact force of 0.5 N, an instrument speed of 1 mm/s, a deformation percentage of 50%, and a recovery distance of 50 mm. The hardness, cohesiveness, springiness, gumminess, and chewiness of the yogurt were determined by measuring the maximum force during sample compression.

#### 2.4.5. Determination of Viscosity

A viscometer (RVDV-II Pro, Brookfield, NY, USA) was employed to determine the viscosity at 25 °C for 60 s at 5 rpm using 63 spindles. The viscosity reading was expressed in centipoises (cP).

### 2.5. Enumeration of the Starter Cultures

Serial dilution and spread plating techniques were employed to determine the microbial viability in the yogurt, which was expressed as CFU/g. Enumeration was performed using MRS agar for *L. bulgaricus* and M17 agar for *S. thermophilus*. *S. thermophilus* was incubated for 72 h at 37 °C in aerobic conditions, while the same parameters were used for *L. bulgaricus* incubation in anaerobic conditions [28].

### 2.6. Free Phenolic Content (FPC) and Antioxidant Properties

#### 2.6.1. Preparation of the Yogurt Extracts

The MFL, MPL, and yogurt samples were extracted using a methanol–acetonitrile solution (1:1), with a sample-to-extraction solution volume ratio of 1:4 and an ultrasonic extraction time of 10 min. The samples were frozen in a refrigerator for 1 h at −20 °C and centrifuged at 2860× *g* for 10 min, after which the supernatant was collected. The extracts were stored at −20 °C until analysis.

#### 2.6.2. Determination of the FPC

The Folin–Ciocalteu method was employed to measure the FPC, and a calibration curve was established using gallic acid as a reference substance to determine polyphenols [29]. Briefly, 0.5 mL of the yogurt extract was mixed with 2.5 mL of Folin–Ciocalteu solution (0.2 mol/L) and incubated in the dark for 5 min at room temperature. Next, 2.0 mL of a 7.5% sodium carbonate solution was added, mixed well, and left to stand in the dark at room temperature for 1 h. The results were expressed as milligrams of GAE per 100 g of yogurt (mg GAE/100 g).

#### 2.6.3. DPPH Radical Scavenging Ability

The DPPH radical scavenging ability was determined using a previously described method with slight modifications [30]. Here, 100 μL of the yogurt extract was mixed with 100 μL of a DPPH solution (0.2 mM, 95% methanol) and incubated in the dark for 30 min at 37 °C. The absorbance was measured at 517 nm, and the DPPH radical scavenging was calculated as follows:

(2) $$\mathrm{Scavenging}\;\mathrm{rate}\;\left(\%\right)=(1\;-\frac{A_{\mathrm{sample}}}{A_{\mathrm{control}}})\times 100$$

where A_sample_ is the absorbance of the yogurt extract and A_control_ is the absorbance of the methanol–acetonitrile solution at a 1:1 volume ratio.

#### 2.6.4. ABTS Radical Scavenging Ability

The ABTS free radical scavenging ability of the yogurt samples was determined using a modified method described by Zhongxiang et al. [31]. First, 88.0 μL of a 140 mM potassium persulfate solution was added to 5.0 mL of a 7 mM ABTS solution and left to stand in the dark for 12–16 h at room temperature to obtain the ABTS radical stock solution. The ABTS radical stock solution was diluted with 70% ethanol so that the absorbance of the diluted ABTS radical stock solution at 734 nm was 0.6–0.7, which was set aside. Then, 3.9 mL of the diluted ABTS radical stock solution with an absorbance of 0.6–0.7 was added to 0.1 mL of the sample solution, mixed well, and reacted in the dark for 6 min at room temperature, after which the absorbance (A_sample_) was measured at 734 nm. Next, 0.1 mL of a methanol–acetonitrile solution at a volume ratio of 1:1 was added to 3.9 mL of the diluted ABTS radical stock solution with an absorbance of 0.6–0.7. The absorbance (A_control_) was measured at 734 nm. The ABTS radical scavenging rate was calculated using the following formula:

(3) $$\mathrm{Scavenging}\;\mathrm{rate}\;\left(\%\right)=(1\;-\frac{A_{\mathrm{sample}}}{A_{\mathrm{control}}})\times 100$$

### 2.7. Anti-Inflammatory Activity

#### 2.7.1. Preparation of the Yogurt Supernatants

A 10 g yogurt sample was centrifuged at 4330× *g* for 5 min at 4 °C to prepare the yogurt supernatant. Then, the supernatants were re-centrifuged in the same conditions and stored at −80 °C until use [32].

#### 2.7.2. Cell Culture

Human epithelial Caco-2 cells (Shanghai Fuheng Biotechnology Co., Ltd., Shanghai, China) were cultivated in Roswell Park Memorial Institute (RPMI)-1640 medium (EallBio, Beijing, China) supplemented with 15% fetal bovine serum (FBS) (Fuheng Biotechnology, Shanghai) and 1% penicillin/streptomycin (EallBio, Beijing, China) and incubated at 37 °C in a humidified chamber with 5% CO_2_. The cells were passaged when they reached 80% confluence.

#### 2.7.3. Real-Time RT-PCR

Caco-2 cells (3 × 10^6^/mL) were inoculated into the cell culture dishes for 24 h until reaching about 60–70% confluence. To induce inflammation, the Caco-2 cells were incubated for 24 h with 2% DSS in FBS-free medium. To study the effect of the millet yogurts, the cells were subsequently treated with yogurt supernatant (100 μL/mL) in FBS-free medium.

Total RNA was extracted using an RNA extraction kit (TransGen Biotech, Beijing, China) according to the instructions. A spectrophotometer was employed to determine the quality and quantity of the total RNA at an absorbance of A_260_/A_280_ nm. The cDNA (complementary DNA) was synthesized from the isolated RNA using a reverse transcription kit (Beyotime, Shanghai, China) and detected using a SYBR Green Master Mix (Toyobo, Shanghai) to determine the TNF-α, IL-6, and IL-1β gene expression via real-time PCR, which used β-actin as an internal reference. The target gene expression was calculated via β-actin normalization using the delta–delta Ct method [33]. The specific primer sequences used are shown in the Supplementary Table S1.

### 2.8. Sensory Evaluation

The sensory evaluation of the yogurt was performed using a method delineated by Li et al. [34], with some modifications. The sensory characteristics of the samples were assessed by 10 trained panelists from the School of Food and Health at Beijing Technology and Business University, who were familiar with yogurt. The panelists consisted of five males and five females aged between 18 and 30 years, with an average age of 24 years. The reviewers successively evaluated six randomly numbered yogurt samples, assessing their appearance, taste, and flavor. Definitions were established for the sensory evaluation terms and scoring criteria. A 10-point intensity scoring system (weak: 0–2, moderate: 3–6, and strong: 7–10) was adopted for each term, as detailed in Table 2. The results were presented in radar charts.

### 2.9. Statistical Analysis

All analyses were repeated three times, and the results were recorded as mean values ± SDs. Significance was determined via one-way analysis of variance (ANOVA) and Duncan’s multiple range tests with a 95% confidence level (*p* < 0.05). SPSS 17.0 (SPSS Inc., Chicago, IL, USA) was used to compare the differences among the values at this significance level.

## 3. Results and Discussion

### 3.1. Characteristics of the Millet Liquid

Table 3 shows the basic nutrient components in the MFL and MPL. The crude fiber was eliminated from the millet during milling, while it was present during pulping and removed during filtration. Therefore, the concentration of the solids and the FPC in the MPL were lower than in the MFL for the same percentage of water added. Previous experiments showed that the soluble solids in the millet increased from 5% to 18.23% after liquefaction by α-amylase. Starch is the primary constituent of millet. Using amylase for gelatinized starch degradation can effectively enhance millet fluidity, promoting its application. Liu et al. found that using heat-resistant α-amylase for steaming at a temperature of 121 °C effectively degraded starch in rice bran via gelatinization and liquefaction [35].

### 3.2. Fermentation Characteristics of the Yogurt

Table 4 shows the fermentation characteristics of the yogurt. A pH of 4.6, which corresponds to the isoelectric point of casein, is crucial in regulating the fermentation endpoint of yogurt, while TA is an important indicator of acid production by lactic acid bacteria during fermentation. The TA of SLY and MCY was significantly higher than that of CY when the pH of the fermentation endpoint was controlled at the same level (*p* < 0.05), while the TA of MFY60 and MPY60 exceeded that of MCY (*p* < 0.05). This was mainly ascribed to higher levels of solids and proteins in the samples, which improved the buffering capacity, necessitating a higher amount of acid to achieve the same pH in the yogurt [36]. Ye et al. demonstrated that with the addition of Tartary buckwheat, the solids content increased and the yogurt titratable acidity increased significantly [11]. The elevated TA of MFY and MPY compared to MCY was attributable to the higher millet protein levels in the former, which enhanced the buffering capacity.

SLY and MCY reached higher acidity levels more rapidly than CY, suggesting faster acid production in the former. This may be related to the presence of glucose since the glucoamylase introduced during fermentation promotes the hydrolysis of small-molecule dextrins and maltodextrins in SLY and MCY, resulting in glucose release. The ability of *Lactobacilli* to utilize monosaccharides more efficiently than disaccharides accelerated acid production [37]. In addition, plant polyphenols may enhance lactic acid bacterial growth and acid production. Kwon [38] and Jeong et al. [39] found that adding chia seed extract or green tea reduced the yogurt fermentation time, suggesting that polyphenols were primarily responsible for this effect.

The *L. bulgaricus* abundance was significantly higher in SLY than in CLY (*p* < 0.05), while no differences were evident in the *S. thermophilus* content (*p* > 0.05), suggesting the presence of components in the millet liquid that specifically promoted *L. bulgaricus* growth. Although many studies have reported that various plant-derived components promote the growth of lactic acid bacteria, the effect varies depending on the plant and the lactic acid bacterial species. Mohamed Ahmed et al. demonstrated that supplementing yogurt with argel leaf extract (ALE) promoted the proliferation of *L. bulgaricus* and *S. thermophilus*, possibly due to the presence of phenolic compounds in the ALE [40]. Ye et al. also found that the addition of Tartary buckwheat to yogurt promoted the growth of *L. bulgaricus* and *S. thermophilus* [11]. However, other studies indicated that incorporating anthocyanin-rich rice or grape pomace and concentrated strawberry pulp into yogurt did not significantly influence lactic acid bacterial growth [12,41,42].

### 3.3. Textural Properties

**Figure 1:**
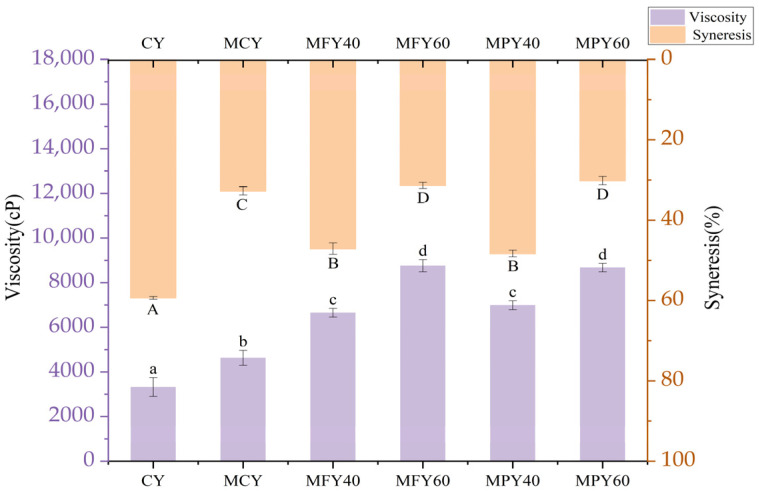
Viscosity and syneresis of the control yogurt and yogurt with millet liquid. Note: Abbreviations: CY: control yogurt; MCY: maltodextrin control yogurt; MFY40/60: 40%/60% millet flour liquid yogurt; MPY40/60: 40%/60% millet paste liquid yogurt. The results are expressed as mean ± SD (n = 3). Means with different superscripts are significantly different (*p* < 0.05).

The structural characteristics of the yogurt were primarily defined according to apparent viscosity, syneresis, and textural properties, such as springiness and hardness, which directly affected product quality. The stability of the yogurt curd was influenced by several factors, such as protein and total solids content, homogenization process, storage conditions, microbial activity, and acidity [26]. Figure 1 shows the apparent viscosity and syneresis of the yogurt. The apparent viscosity of SLY and MCY was significantly higher (*p* < 0.05) than CY, while the syneresis was substantially lower (*p* < 0.05), possibly due to the increase in the total solids content. The apparent viscosity of MFY60 and MPY60 displayed a significant increase (*p* < 0.05) while the syneresis was significantly decreased (*p* < 0.05) compared to MCY with the same solids content, possibly due to the presence of polyphenols and dietary fibers in the millet liquid, which yielded a stronger gel network structure. Mohamed Ahmed et al. indicated that incorporating ALE enhanced the viscosity of yogurt and reduced syneresis, likely attributable to the interaction between proteins and polyphenols. This facilitated the development of stronger three-dimensional networks and viscous gels that could retain larger amounts of whey [40]. In addition, Ramandeep [9] and Qu et al. [10] demonstrated that adding soluble dietary fiber improved yogurt viscosity and syneresis. Since the soluble dietary fiber exhibited a significant water-holding capacity, its interaction with casein resulted in highly viscous yogurt that effectively retained water and prevented whey precipitation.

Table 5 shows the effect of the millet liquid on the textural properties of the yogurt. The results showed that SLY displayed significantly more springiness and chewiness (*p* < 0.05) than CLY, while no statistical differences (*p* > 0.05) were evident in the hardness and cohesiveness. Mohamed Ahmed et al. found that the addition of ALE significantly increased the springiness of yogurt and attributed this to the interaction between the phenolic compounds in the ALE and the yogurt proteins [40]. Rose Mary reported that yogurt fortified with dietary fiber yielded higher springiness values [43]. Therefore, the improved textural properties of the yogurt, such as springiness and chewiness, may be attributed to the interaction of phenolic compounds and dietary fiber with the casein in the millet liquid, resulting in the formation of a more stable gel network structure. Polyphenols have reactive hydroxyl and carboxylic acid groups that bind to casein through non-covalent interactions (e.g., hydrophobic interactions, hydrogen bonding). In particular, the hydrophobic portion of polyphenols binds to the hydrophobic regions of casein and promotes cross-linking of the protein to form a denser gel network. In addition, the hydroxyl groups of polyphenols form hydrogen bonds with the polar groups (e.g., amino and carboxyl groups) of casein to enhance network strength [44]. Dietary fibers (e.g., soluble arabinoxylan) have strong water retention properties and form weak interactions (e.g., hydrophobic interactions) with casein to influence the gel network structure [9]. Thus, polyphenols and fibers directly optimize the cross-linking density and water-holding properties of the casein network through the above mechanisms, resulting in selective improvement in springiness and chewability. Studies have shown that the protein matrix in flaxseed represents the primary factor in enhancing the cohesiveness of yogurt [45]. However, the protein content of the millet liquid in the present study was insufficient to influence cohesiveness.

### 3.4. Sensory Analysis

**Figure 2:**
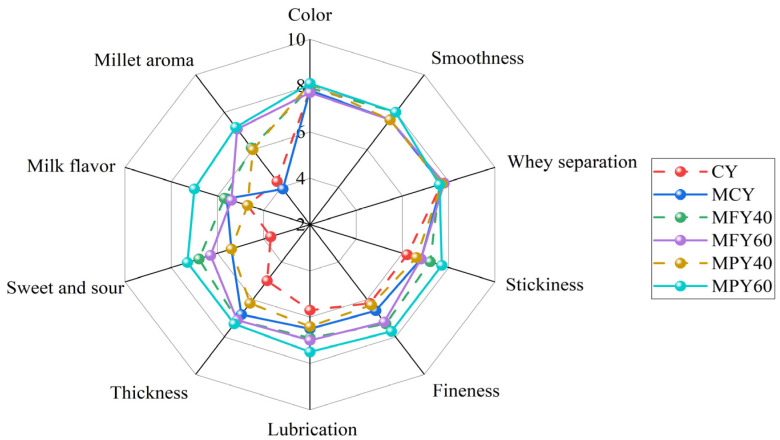
Sensory evaluation of the control yogurt and the yogurt with millet liquid. Note: Abbreviations: CY: control yogurt; MCY: maltodextrin control yogurt; MFY40/60: 40%/60% millet flour liquid yogurt; MPY40/60: 40%/60% millet paste liquid yogurt.

Figure 2 shows the sensory evaluation results of the yogurt. In terms of taste, MCY and SLY scored significantly higher in thickness and sweet and sour taste compared to CY (*p* < 0.05). The enhancement in yogurt thickness with the addition of maltodextrin or millet liquid was consistent with the significant increase in apparent viscosity, which was associated with an increase in the solids content. Previous research showed that adding oat fiber improved the taste of unsweetened yogurt, with the proposed mechanism involving a higher total solids content [46]. During fermentation, the millet liquid or maltodextrin released glucose in the presence of glucoamylase, which increased the sweetness of the yogurt and improved its taste. In terms of flavor, there was no significant difference in the scores for the milk flavor of the yogurt with millet liquid compared to the control samples, but the scores for millet aroma were significantly higher. These results suggest that millet contributes a distinctive flavor profile to yogurt while maintaining the inherent milk flavor, indicating potential for improved consumer appeal. Differences in yogurt aroma are related to the formation of volatile compounds. The main volatile compounds in fermented millet beverages include benzene derivatives, hexadecanoic acid, acetic acid, sitosterol, and octadecanoic acid [47]. The formation of desirable flavors in yogurts containing cereal and milk may be related to the presence of phenolic compounds and the volatile phenolics they produce [29,48]. No significant differences were evident between the scores of the yogurt groups in terms of appearance (*p* > 0.05). The addition of millet liquid did not affect the color scores of the yogurt since the liquid was light beige and closely resembled the color of milk.

### 3.5. The FPC and Antioxidant Activity in the Yogurt

**Figure 3:**
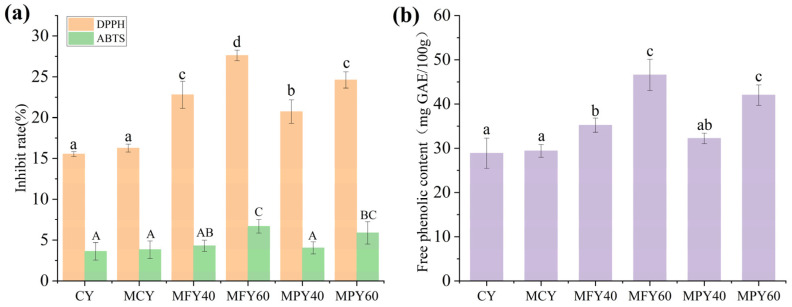
(**a**) DPPH, ABTS radical scavenging activities, and (**b**) free phenolic content of the control yogurt and the yogurt with millet liquid. Note: Abbreviations: CY: control yogurt; MCY: maltodextrin control yogurt; MFY40/60: 40%/60% millet flour liquid yogurt; MPY40/60: 40%/60% millet paste liquid yogurt. The results are expressed as mean ± SD (n = 3). Means with different superscripts are significantly different (*p* < 0.05).

The in vitro antioxidant activity of the millet yogurt was assessed by examining the DPPH and ABTS radical scavenging capacity (Figure 3a). Compared with the CLY group, the yogurt samples with millet liquid exhibited a higher DPPH radical scavenging ability, with the MFY group surpassing the MPY group. Only the yogurt containing 60% millet liquid exhibited enhanced ABTS radical scavenging ability, with no discernible difference between the MPY and MFY groups. This suggests that the increased antioxidant capacity of blended millet yogurt depends on the quantity of added millet and the treatment applied and that it is related to the FPC. The FPC of the MY group exceeded that of the CLY group, with MY60 displaying a 40% higher level than the CLY group (Figure 3b). The elevated antioxidant activity of MFY compared to MPY may also be attributed to its superior FPC, despite the lack of substantial statistical differences. Demirci et al. investigated the effect of tomato powder on yogurt. They found that with an increase in the tomato powder content, the results of DPPH/ABTS radical scavenging ability tests were positively correlated with the FPC concentration [49]. The increased antioxidant activity of the yogurt could also be related to other antioxidants in the millet, such as carotenoids, α-tocopherol, minerals, and several polysaccharides [15,50]. These findings indicated that adding millet liquid to the yogurt enhanced the bioactive properties and antioxidant activity, consequently improving the potential health benefits.

The MFY60 and MPY60 displayed FPC levels of 46.58 mg GAE and 42.01 mg GAE, respectively (Figure 3b), both of which were lower the sum of the FPC in the 60% millet liquids (26.78 mg GAE and 24.42 mg GAE) and the CY (28.85 mg GAE). Also the results revealed that the FPC before and after fermentation was 46.71 mg GAE and 35.20 mg GAE in MFY40 and 45.13 mg GAE and 32.22 mg GAE in MPY40. Thus, the results suggest that fermentation may contribute to the conversion of free phenols to bound phenols in millet liquid. It is hypothesized that this may be related to some aggregation of milk proteins with free phenols from the millet liquid during fer-mentation.

### 3.6. Anti-Inflammatory Activity of the Millet Yogurt

**Figure 4:**
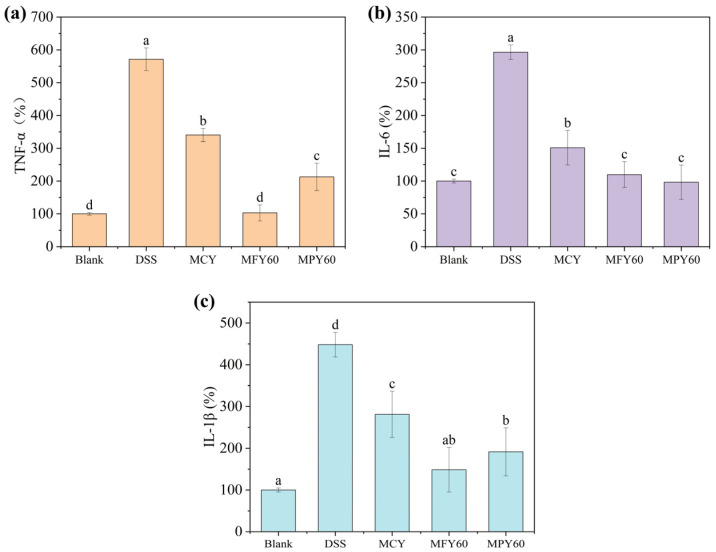
Effects of the yogurt with 60% filtered millet liquid on DSS-induced inflammation in Caco-2 cells. (**a**) TNF-α; (**b**) IL-6; (**c**) IL-1β. Note: Abbreviations: MCY: maltodextrin control yogurt; MFY 60: 60% millet flour liquid yogurt; MPY 60: 60% millet paste liquid yogurt. Data are presented as mean ± SD (n = 3). The values with different superscript letters are significantly different (*p* < 0.05).

Studies have shown that a variety of polyphenol-rich plant-derived extracts and phenolic compounds mitigate inflammation [51]. Therefore, the anti-inflammatory activity of MFY60 and MPY60 was further assessed by examining the effect of the yogurt supernatant on DSS-induced inflammatory factor expression in Caco-2 cells. The results (Figure 4) showed that 2% DSS treatment significantly increased the mRNA expression levels of TNF-α, IL-1β, and IL-6 compared to the blank control (*p* < 0.05), which was substantially inhibited by the addition of all three yogurt supernatant samples (*p* < 0.05). The incorporation of MFY60 and MPY60 markedly downregulated TNF-α, IL-6, and IL-1β expression compared to MCY (*p* < 0.05). The addition of MFY60 yielded the most significant alleviation effect, almost reaching the level of the blank group. Elevated TNF-α, IL-6, and IL-1β levels are closely associated with various inflammatory and autoimmune diseases [52]. The addition of millet may mitigate the impact of colitis by inhibiting inflammatory cytokine expression. A recent study demonstrated that millet-derived polyphenols alleviate DSS-induced colitis through dual mechanisms: (1) enhancing intestinal barrier integrity by upregulating tight junction-associated proteins (Claudin-1, ZO-1, and Occludin) and mucin family genes (e.g., MUC2) while increasing goblet cell density to fortify the mucus layer, and (2) modulating gut microbiota homeostasis via the selective inhibition of pro-inflammatory bacterial taxa (e.g., S24-7 and Staphylococcaceae) and promoting commensal bacteria associated with anti-inflammatory responses (e.g., Lachnospiraceae and Rikenellaceae) [53].

## 4. Conclusions

This study shows that yogurt supplemented with millet displays superior sensory attributes and functional properties. The addition of millet liquid solids and phenolic and protein components can specifically stimulate the growth of *L. bulgaricus* in yogurt. This improves its viscosity and textural properties (springiness and chewiness) and decreases syneresis. Meanwhile, the yogurt with millet liquid may become popular among consumers because of the high-scored taste and aroma in comparison to the control sample. In addition, incorporating millet liquid into yogurt presents significant potential health benefits. The combined fermentation of millet liquid and yogurt enhances the DPPH and ABTS free radical scavenging capacity, exhibiting antioxidant activity. The supernatant of MY60 significantly alleviates the expression of the TNF-α, IL-1β, and IL-6 pro-inflammatory cytokines produced by cells stimulated by DSS, consequently demonstrating anti-inflammatory activity. This study provides a reference for developing functional yogurt with favorable physicochemical properties, antioxidant and anti-inflammatory activity, and organoleptic characteristics. Future mechanistic research can focus on using millet to improve the anti-inflammatory activity of yogurt.

## Figures and Tables

**Table 1:** Formulations of the control yogurt and the yogurt with millet liquid.

Group	Item	Distilled Water (g)	Millet Liquid (g)	Whole Milk Powder (g)	Maltodextrin (g)	Glucoamylase (μL)	Starter Culture (g)
Control groups (CLY)	Control yogurt (CY)	500	-	62	-	-	0.05
	Maltodextrin control yogurt (MCY)	440	-	62	60	48	0.05
Sample groups (SLY)	40% Millet flour liquid yogurt (MFY40)	300	200	62	-	32	0.05
	60% Millet flour liquid yogurt (MFY60)	200	300	62	-	48	0.05
	40% Millet paste liquid yogurt (MPY40)	300	200	62	-	32	0.05
	60% Millet paste liquid yogurt (MPY60)	200	300	62	-	48	0.05

**Table 2:** Sensory evaluation scoring index and criteria of the yogurt samples.

Sensory Property		Definition of Evaluation Terms and Scoring Criteria		
		0–2	3–6	7–10
Appearance	Color	Uneven color, dull color	Uneven color, overall pale yellow or creamy white	Uniform and consistent color, overall creamy white or creamy yellow
	Smoothness	The surface is not smooth	The surface is generally smooth	Smooth surface
	Whey separation	Massive whey separation	Small amount of whey separation	No whey separation
	Stickiness	Fragile structure, high fluidity	Not loosely structure, general fluidity	Tight structure, low fluidity
	Fineness	Rough structure	Fine structure	Fine and uniform structure
Taste	Lubrication	Clearly grainy	Slightly grainy, not lubricated	No grainy feeling, lubricated
	Thickness	Very thin	General	Mellow
	Sweet and sour	Too sweet or too sour	Moderately sweet and sour, slightly astringent	Suitable sour and sweet taste
Flavor	Milk flavor	Light yogurt flavor	Moderate yogurt flavor	Typical yogurt flavor
	Millet aroma	Poor characteristic flavor of added millet	Insufficient characteristic flavor of added millet	Strong characteristic flavor of added millet

**Table 3:** Chemical composition of filtered millet liquid.

Parameters	Total Solids (g/100 g)	Protein (g/100 g)	Soluble Solids (g/100 g)	Free Phenol (mg GAE/100 g)
MFL	21.11 ± 0.12	0.15 ± 0.00	20.2 ± 0.22	44.64 ± 2.58
MPL	19.19 ± 0.12	0.14 ± 0.01	18.23 ± 0.20	40.70 ± 1.32

Note: Abbreviations: MFL: millet flour liquid; MPL: millet pulp liquid. The results are expressed as mean ± SD (n = 3).

**Table 4:** pH values, titrable acidity, fermentation time, and lactic acid bacteria count of the control yogurt and the yogurt with millet liquid.

Group	Sample	pH	T_f_ (h)	Titratable Acidity (%)	*S. thermophilus* (Log CFU/g)	*L. bulgaricus* (Log CFU/g)
CLY	CY	4.54 ± 0.01 ^a^	4.07 ± 0.13 ^d^	0.72 ± 0.01 ^a^	8.33 ± 0.02 ^a^	8.26 ± 0.02 ^a^
	MCY	4.53 ± 0.01 ^a^	3.60 ± 0.11 ^c^	0.88 ± 0.01 ^b^	8.34 ± 0.03 ^a^	8.24 ± 0.05 ^a^
SLY	MFY40	4.52 ± 0.01 ^a^	3.18 ± 0.11 ^a^	0.91 ± 0.01 ^c^	8.34 ± 0.02 ^a^	8.58 ± 0.03 ^b^
	MFY60	4.53 ± 0.01 ^a^	3.35 ± 0.06 ^ab^	0.96 ± 0.01 ^d^	8.35 ± 0.02 ^a^	8.58 ± 0.01 ^b^
	MPY40	4.54 ± 0.02 ^a^	3.22 ± 0.11 ^a^	0.91 ± 0.01 ^c^	8.34 ± 0.01 ^a^	8.55 ± 0.06 ^b^
	MPY60	4.54 ± 0.02 ^a^	3.46 ± 0.13 ^bc^	0.97 ± 0.02 ^d^	8.33 ± 0.04 ^a^	8.57 ± 0.05 ^b^

Note: Abbreviations: CLY: control groups; SLY: sample groups; CY: control yogurt; MCY: maltodextrin control yogurt; MFY40/60: 40%/60% millet flour liquid yogurt; MPY40/60: 40%/60% millet paste liquid yogurt; Tf: time to complete the fermentation at pH 4.6. The results are expressed as mean ± SD (n = 3). Means with different superscripts are significantly different (*p* < 0.05).

**Table 5:** Texture characteristics of the control yogurt and the yogurt with millet liquid.

Group	Sample	Hardness (N)	Cohesiveness (Ratio)	Springiness (mm)	Gumminess (N)	Chewiness (mj)
CLY	CY	0.60 ± 0.03 ^a^	0.82 ± 0.03 ^a^	0.15 ± 0.02 ^a^	0.45 ± 0.00 ^a^	0.81 ± 0.02 ^a^
	MCY	0.61 ± 0.00 ^a^	0.88 ± 0.04 ^a^	0.54 ± 0.17 ^a^	0.52 ± 0.01 ^bc^	0.30 ± 0.07 ^a^
SLY	MFY40	0.57 ± 0.03 ^a^	0.89 ± 0.02 ^a^	9.85 ± 0.06 ^b^	0.51 ± 0.01 ^b^	4.32 ± 0.31 ^b^
	MFY60	0.63 ± 0.28 ^a^	0.89 ± 0.02 ^a^	7.83 ± 0.28 ^c^	0.53 ± 0.01 ^cd^	4.40 ± 0.14 ^b^
	MPY40	0.61 ± 0.01 ^a^	0.83 ± 0.04 ^a^	9.68 ± 0.39 ^b^	0.51 ± 0.01 ^bc^	4.52 ± 0.11 ^b^
	MPY60	0.65 ± 0.02 ^a^	0.82 ± 0.04 ^a^	8.38 ± 0.87 ^c^	0.54 ± 0.01 ^d^	4.52 ± 0.43 ^b^

Note: Abbreviations: CLY: control groups; SLY: sample groups; CY: control yogurt; MCY: maltodextrin control yogurt; MFY40/60: 40%/60% millet flour liquid yogurt; MPY40/60: 40%/60% millet paste liquid yogurt. The results are expressed as mean ± SD (n = 3). Means with different superscripts are significantly different (*p* < 0.05).

## Data Availability

The original contributions presented in this study are included in the article and Supplementary Materials; further inquiries can be directed to the corresponding authors.

## Supplementary Materials

The following supporting information can be downloaded at https://www.mdpi.com/article/10.3390/foods14203491/s1: Table S1: Primer sequences of RT-qPCR.
