# Supplementary material for: The Physicochemical, Sensory, and Functional Properties of Yogurt Containing Millet and Milk
Source: Foods. 2025 Oct 14;14(20):3491. doi: 10.3390/foods14203491 (PMC12564760; doi:10.3390/foods14203491)
Supplement: Supplementary file 1 [file foods-14-03491-s001.zip › foods-3610102-supplementary.pdf]

Supplementary Tables

Supplementary Table S1 Primer sequences of RT-qPCR.

**Gene**	**Primer sequences**
β-actin-F	CCTAGAAGCATTTGCGGTGCACGATG
β-actin-R	TCATGAAGTGTGACGTTGACATCCGT
TN-α-F	TGGCGTGGAGCTGAGAGATAACC
TNF-α-R	GACGGCGATGCGGCTGATG
IL-6-F	CACTGGTCTTTTGGAGTTTGAG
IL-6-R	GGACTTTTGTACTCATCTGCAC
IL-1β-F	CTGTAGTGGTGGTCGGAGATTCG
IL-1β-R	CAGTGGCAATGAGGATGACTTGTTC
